# Supplementary material for: Redefining topological robustness in optical polarization fields through a generalized skyrmion number
Source: Nat Commun. 2026 Jul 10;17:8539. doi: 10.1038/s41467-026-75120-7 (PMC13483919; doi:10.1038/s41467-026-75120-7)
Supplement: Supplementary file 1 [file 41467_2026_75120_MOESM1_ESM.pdf]

Supplementary Information
Redefining topological robustness in optical polarization fields through a generalized skyrmion number

An Aloysius Wang[1,*], Yifei Ma[1], Zimo Zhao[1], Yuxi Cai[1], Stephen M Morris[1], Honghui He[2], Zhenwei Xie[3], Peng Shi[3], Yijie Shen[4], Anatoly V Zayats[5], Xiaocong Yuan[3], and Chao He[1,*]

[1]Department of Engineering Science, University of Oxford, Parks Road, Oxford, OX1 3PJ, UK
[2]Guangdong Research Center of Polarization Imaging and Measurement Engineering Technology, Tsinghua Shenzhen International Graduate School, Tsinghua University, Shenzhen 518055, China
[3]Nanophotonics Research Center, Shenzhen Key Laboratory of Micro-Scale Optical Information Technology & Institute of Microscale Optoelectronics, Shenzhen University, Shenzhen 518060, China
[4]Division of Physics and Applied Physics, School of Physical and Mathematical Sciences, Nanyang Technological University, Singapore, Singapore
[5]Department of Physics and London Centre for Nanotechnology, King's College London, London, UK
[*]Corresponding authors: aloysius.wang@gmail.com; chao.he@eng.ox.ac.uk

1 Supplementary Note

1.1 Defining the Generalized Skyrmion Number

In this admittedly long and technical section, we build up to the definition of the generalized skyrmion number in three steps. To facilitate our discussion, we begin by providing a brief overview of our approach, including key terminology and notation. In this section, we consider smooth fields $\mathcal{S}\colon \Omega \longrightarrow Y$ where Ω is an open subset of $\mathbb{R}^n$ with the usual orientation and Y a compact, connected, oriented, smooth, n-dimensional manifold. When specializing our theory to optical polarization fields, we will explicitly state so, and make clear that, in fact, $Y = S^2$. Lastly, we denote the open ball of radius r centered about $p \in \mathbb{R}^n$ by $B_r(p) \subset \mathbb{R}^n$.

Our three main steps are as follows: Firstly, we generalize the compactification process introduced in [1] to fields taking values in an arbitrary manifold Y and define the notion of generalized Y-valued skyrmions for which the usual skyrmion number integral is guaranteed to be integer-valued.

Secondly, we consider the case where fields are no longer compactifiable, and for each normalized $\omega \in \Lambda^n(Y)$, assign to the possibly non-compactifiable field $\mathcal{S}$ a corresponding ω-skyrmion number. Next, by replacing compactifiability with the more general notion of (ψ, F)-extendability, we recover some way of interpreting the ω-skyrmion number topologically.

Thirdly, by examining the qualitative behavior of the ω-skyrmion number as ω varies across $\Lambda^n(Y)$, we show that for each $\mathcal{S}$, it is possible to partition $\Lambda^n(Y)$ into collections of forms for which the corresponding notion of ω-skyrmion number is consistent and integer-valued. Each partition, which can be associated with a connected component carved out by the boundary of the field, then defines a single topological number. We then formalize this observation by turning to the de Rham cohomology of compactly supported forms [2]. Lastly, we provide a method of establishing the topological robustness of the generalized skyrmion number against deformations of the field using appropriate (ψ, F)-extensions.

In each of the three steps, we prove an abstract result of topological protection relevant to the generality of the skyrmion number defined in that section, and highlight these results as **Theorems**. We then ground our abstract results in reality by providing a description of how they can be applied to optical polarization fields.

Lastly, in our exposition of the topic, we will, at times, be loose with distinctions between smoothness and continuity, noting that appropriate cohomology theories can stand in for the de Rham cohomology to make everything rigorous (see Supplementary Note 2).

1.2 Y-Valued Skyrmions

We briefly recall the notion of compactifiability defined in [1]. Let $\mathcal{S}\colon \Omega \longrightarrow Y$ be a smooth map. Suppose there exists a compact, connected, oriented, smooth n-dimensional manifold X and a smooth map $\psi\colon \Omega \longrightarrow X$ such that

1. ψ is an orientation preserving diffeomorphism of Ω onto its image,
2. $\psi(\Omega)$ is a dense subset of X with full measure,
3. $\mathcal{S} \circ \psi^{-1}$ extends via continuity to a smooth map $\tilde{\mathcal{S}}$ on all of X,

then we say $\mathcal{S}$ is ψ-compactifiable, and call $\tilde{\mathcal{S}}$ the ψ-compactification of $\mathcal{S}$. If $\mathcal{S}$ is ψ-compactifiable, we define its skyrmion number by

$$\deg \mathcal{S} \coloneqq \deg \tilde{\mathcal{S}},$$

where the deg on the right-hand side of the equation is the usual degree arising from de Rham cohomology [3]. The topological robustness of the skyrmion then arises from the homotopy invariance of the degree, where given fields $\mathcal{S}_1$ and $\mathcal{S}_2$ which are ψ_1- and ψ_2-compactifiable, respectively, with $\operatorname{codom} \psi_1 = \operatorname{codom} \psi_2$, $\deg \mathcal{S}_1 = \deg \mathcal{S}_2$ whenever $\tilde{\mathcal{S}}_1$ and $\tilde{\mathcal{S}}_2$ are smoothly homotopic. If, further, $X = Y = S^n$, we have, via the Hopf degree theorem, the converse statement that whenever $\deg \mathcal{S}_1 = \deg \mathcal{S}_2$, $\tilde{\mathcal{S}}_1$ and $\tilde{\mathcal{S}}_2$ are smoothly homotopic. With the machinery above, we trivially have the following result.

Theorem 1. *Let Ω_z be an open subset of $\mathbb{R}^n$ for each $z \in [z_0, z_f]$ and $\mathcal{S}^z\colon \Omega_z \longrightarrow Y$ a smooth map. Suppose, for each $z \in [z_0, z_f]$, $\mathcal{S}^z$ is compactifiable via $\psi_z\colon \Omega_z \longrightarrow X$. If further $H\colon X \times [z_0, z_f] \longrightarrow Y$, $H(x, z) = \tilde{\mathcal{S}}^z(x)$ is smooth, then $\deg \mathcal{S}^{z_1} = \deg \mathcal{S}^{z_2}$ for all $z_1, z_2 \in [z_0, z_f]$.*

Specializing to optical polarization fields, the theorem above makes rigorous the claim in Methods 1 of topological protection for fields whose transverse polarization profile remains compactifiable in propagation. In the case of vector beams formed from a superposition of LG modes, the polarization state at infinity depends on the asymptotic behavior of the individual modes. If, for example, a single mode dominates in the far field, and provided there are no polarization singularities, taking $\Omega_z = \mathbb{R}^2$ and ψ_z to be any inverse stereographic projection establishes topological protection by the theorem presented above.

1.3 The ω-Skyrmion Number and (ψ, F)-Extensions

Without compactifiability, the usual skyrmion number integral is no longer guaranteed to be the same for all normalized $\omega \in \Lambda^n(Y)$. Nevertheless, we can still define, for each normalized $\omega \in \Lambda^n(Y)$, a form-dependent skyrmion number

$$\mathrm{SkyN}_\omega(\mathcal{S}) \coloneqq \int_\Omega \mathcal{S}^*\omega,$$

which we call the ω-skyrmion number of $\mathcal{S}$. To interpret ω-skyrmion numbers topologically, we introduce the following extension process. Let X be a compact, connected, oriented, smooth n-dimensional manifold and $\psi\colon \Omega \longrightarrow X$ be an orientation preserving diffeomorphism onto its image. We say $\mathcal{S}$ is ψ-extendable if

1. $\mathcal{S} \circ \psi^{-1}$ extends via continuity to $\overline{\psi(\Omega)}$ and
2. there exists a smooth $F\colon X - \psi(\Omega) \longrightarrow Y$ such that $F|_{\partial\psi(\Omega)} = \mathcal{S} \circ \psi^{-1}|_{\partial\psi(\Omega)}$, where the latter is to be understood as the continuous extension of $\mathcal{S} \circ \psi^{-1}$ to $\overline{\psi(\Omega)}$.

We then call

$$\tilde{\mathcal{S}}_F = \begin{cases} \mathcal{S} \circ \psi^{-1}, & x \in \overline{\psi(\Omega)} \\ F, & x \in X - \psi(\Omega) \end{cases}$$

the (ψ, F)-extension of $\mathcal{S}$ to X, where $\tilde{\mathcal{S}}_F$ is continuous by a gluing argument and piecewise smooth by construction.

Ignoring technicalities arising from piecewise smoothness, we can relate $\mathrm{SkyN}_\omega(\mathcal{S})$ to the degree of the map $\tilde{\mathcal{S}}_F$ via the equation

$$\deg \tilde{\mathcal{S}}_F = \mathrm{SkyN}_\omega(\mathcal{S}) + \int_{X-\psi(\Omega)} F^*\omega. \tag{1}$$

Notice further that whenever $\mathcal{S}$ is ψ-compactifiable, $\mathrm{SkyN}_\omega(\mathcal{S}) = \deg \mathcal{S}$ for all normalized $\omega \in \Lambda^n(Y)$ as the density of $\psi(\Omega)$ in X implies that $\mathcal{S}$ extends to a unique $\tilde{\mathcal{S}}_F = \tilde{\mathcal{S}}$.

We now state our main topological robustness result for ω-skyrmion numbers.

Theorem 2. Let Ω_z be an open subset of $\mathbb{R}^n$ for each $z \in [z_0, z_f]$ and $\mathcal{S}^z\colon \Omega_z \longrightarrow Y$ a smooth map. Suppose, for each $z \in [z_0, z_f]$, $\mathcal{S}^z$ is ψ_z-extendable, $\psi_z(\Omega_z)$ is the same for all z, and $J\colon \psi_z(\Omega_z)\times[z_0, z_f] \longrightarrow Y$, $J(x, z) = \mathcal{S}^z(\psi_z^{-1}(x))$ is smooth. If further $\mathcal{S}^z$ when extended to $\overline{\psi_z(\Omega_z)}$ restricts to the same function $\partial\mathcal{S}$ on $\partial\psi_z(\Omega_z)$ for all z, then $\mathrm{SkyN}_\omega(\mathcal{S}^{z_1}) = \mathrm{SkyN}_\omega(\mathcal{S}^{z_2})$ for all normalized $\omega \in \Lambda^n(Y)$, and $z_1, z_2 \in [z_0, z_f]$.

Proof: Any F that extends $\mathcal{S}^z$ for some z must necessarily also extend every other $\mathcal{S}^{z'}$, $z' \in [z_0, z_f]$. Picking one such F, which exists by assumption, set $H\colon X \times [z_0, z_f] \longrightarrow Y$ by $H(x, z) = \tilde{\mathcal{S}}^z_F(x)$. Notice that H is a homotopy by the smoothness of J. The result then follows immediately from the homotopy invariance of the degree and equation (1). ■

As a concrete application of the above to optical polarization fields, consider the situation of a cylindrical conducting waveguide of radius a aligned parallel to the z-axis. Suppose we have a monochromatic wave of the form

$$\boldsymbol{E}(x, y, z, t) = \boldsymbol{E}_0(x, y, z)e^{-\mathrm{i}\omega t}$$
$$\boldsymbol{B}(x, y, z, t) = \boldsymbol{B}_0(x, y, z)e^{-\mathrm{i}\omega t}$$

confined within the conductor. The conductor then imposes the following boundary conditions: the parallel component of the electric field at the air-conductor interface $\boldsymbol{E}_\parallel = \boldsymbol{0}$, and the perpendicular component of the magnetic field at the air-conductor interface $B_\perp = 0$. Crucially, the boundary condition on the electric field implies that

$$\boldsymbol{E}_0(a\cos\theta, a\sin\theta, z) \propto \begin{pmatrix}\cos\theta\\ \sin\theta\\ 0\end{pmatrix}$$

for any z. Suppose further that

$$\|E_x(x, y, z) + E_y(x, y, z)\| > 0$$

for all $(x, y) \in \overline{B_a(0)}$ and $z \in [z_0, z_f]$, then, for each z, the transverse components of the electric field define for us a corresponding Stokes field $\mathcal{S}^z\colon \overline{B_a(0)} \longrightarrow S^2$ that satisfies

$$\mathcal{S}^z(a\cos\theta, a\sin\theta) = \begin{pmatrix}\cos 2\theta\\ \sin 2\theta\\ 0\end{pmatrix}.$$

To extend the field, let ψ be the orientation preserving diffeomorphism

$$\psi(r, \theta) = \left(r\cos\theta, r\sin\theta, \sqrt{1-r^2}\right) \tag{2}$$

that maps $B_a(0)$ onto the open upper hemisphere $S^+ = \{(x^1, x^2, x^3) \in S^2 \colon x^3 > 0\}$ of the Poincaré sphere. Taking $S^- = \{(x^1, x^2, x^3) \in S^2 \colon x^3 < 0\}$, we may construct a distinguished $F\colon S^- \longrightarrow S^2$ given by

$$F\left(\sqrt{1-x_3^2}\cos\theta, \sqrt{1-x_3^2}\sin\theta, x_3\right) = \left(\sqrt{1-x_3^2}\cos 2\theta, \sqrt{1-x_3^2}\sin 2\theta, x_3\right)$$

that canonically extends every such $\mathcal{S}^z$.

Setting $H\colon S^2 \times [z_0, z_f] \longrightarrow S^2$ as $H(x,z) = \tilde{\mathcal{S}}_F^z(x)$, we have a well-defined homotopy from $\tilde{\mathcal{S}}_F^{z_0}$ to $\tilde{\mathcal{S}}_F^{z_f}$. From H, we conclude that

$$\mathrm{SkyN}_\omega(\mathcal{S}^{z_0}) = \mathrm{SkyN}_\omega(\mathcal{S}^{z_f})$$

for all normalized $\omega \in \Lambda^2(S^2)$.

1.4 The Generalized Skyrmion Number

Building on the theory of (ψ, F)-extensions introduced in the previous section, we begin with the following observation. Suppose $\mathcal{S}$ has a (ψ, F)-extension where F is not surjective. Then, any normalized $\omega \in \Lambda^n(Y)$ whose support $\operatorname{supp}\omega \subset Y - F(X - \psi(\Omega))$ satisfies the relation

$$\deg \tilde{\mathcal{S}}_F = \mathrm{SkyN}_\omega(\mathcal{S}) + \int_{X-\psi(\Omega)} F^*\omega = \mathrm{SkyN}_\omega(\mathcal{S}).$$

Therefore, for every ω with the stated restriction, the corresponding ω-skyrmion number is integer-valued. Moreover, any two forms sharing this restriction have the same ω-skyrmion number. This suggests that given a field $\mathcal{S}$, one way to define a generalized skyrmion number is based on the largest suitable subset of forms for which the corresponding notion of ω-skyrmion number is consistent and integer-valued.

To make this more precise, consider the following implication of the observation above. For ease of visualization, let $\mathcal{S}\colon B_1(0) \subset \mathbb{R}^2 \longrightarrow S^2$ and suppose $\mathcal{S}$ extends via continuity to $\overline{B_1(0)}$. Then $\partial\mathcal{S} := \mathcal{S}|_{\partial B_1(0)}$ traces out a curve in S^2 that partitions it into connected components $V_1, \dots, V_k$. Since $\partial B_1(0)$ is compact, $\partial\mathcal{S}(\partial B_1(0))$ is also compact, and hence closed. This then implies that the V_is are open sets. Let $p \in V_i$. By the openness of V_i, there is some $B_r(0) \subset \mathbb{R}^3$ so that $p \in B_r(0) \cap S^2 = U_i \subset V_i$. Consider now the following (ψ, F)-extension. Let ψ be as defined in equation (2) and $F\colon S^- \longrightarrow S^2$ be

$$F\left(\sqrt{1-x_3^2}\cos\theta, \sqrt{1-x_3^2}\sin\theta, x_3\right) = \frac{(1+x_3)\partial\mathcal{S}(\theta) + x_3 p}{\|(1+x_3)\partial\mathcal{S}(\theta) + x_3 p\|}. \tag{3}$$

Then, by construction, $F(S^-) \cap U_i = \varnothing$. Therefore, for any normalized ω with compact support in U_i, $\mathrm{SkyN}_\omega(\mathcal{S})$ is consistent and integer-valued. Since the point p was arbitrary, and V_i is connected, one can essentially regard $\mathrm{SkyN}_\omega(\mathcal{S})$ as a topological number associated with V_i rather than ω.

To formalize this intuition, we review some basic differential geometry. Let X be an n-dimensional smooth manifold. We define by $\Lambda_c^k(X)$ the smooth real-valued k-forms on X with compact support. Then the exterior differentiation maps $d^k\colon \Lambda_c^k(X) \longrightarrow \Lambda_c^{k+1}(X)$ form a cochain complex

$$\cdots \to 0 \to \Lambda_c^0(X) \xrightarrow{d^0} \Lambda_c^1(X) \xrightarrow{d^1} \cdots \xrightarrow{d^{n-1}} \Lambda_c^n(X) \to 0 \to \cdots$$

from which we may define the de Rham cohomology groups with compact supports [2],

$$H_c^k(X) := \ker d^k / \operatorname{im} d^{k+1}.$$

Let $f\colon X \longrightarrow Y$ be a smooth map. We call f proper if for every compact set $L \subseteq Y$, $K = f^{-1}(L)$ is compact. Then, for each $k \in \mathbb{Z}$ and proper smooth map $f\colon X \longrightarrow Y$, we have a natural corresponding map in cohomology

$$f^{\#}\colon H_c^k(Y) \longrightarrow H_c^k(X), \quad [\omega] \mapsto [f^*\omega].$$

One may show that for any connected, oriented, smooth, n-dimensional manifold, $H_c^n(X) \cong \mathbb{R}$ via the isomorphism $\omega \mapsto \int_X \omega$. Therefore, any proper smooth map $f\colon X \longrightarrow Y$ between connected, oriented, smooth, n-dimensional manifolds induces a linear map

$$f^{\#}\colon H_c^n(Y) \cong \mathbb{R} \longrightarrow H_c^n(X) \cong \mathbb{R},$$

which we may identify with a real number $c \in \mathbb{R}$ called the degree of f. As with the degree that originates from the de Rham cohomology, one can show that the degree originating from the de Rham cohomology

of compactly support forms is also integer-valued. From this, we now have all the necessary ingredients to assemble the definition of the generalized skyrmion number.

As before, let Ω be an open subset of $\mathbb{R}^n$, Y a compact, connected, oriented, smooth n-dimensional manifold, and $\mathcal{S}\colon \Omega \longrightarrow Y$ a smooth map. Let $\psi\colon \Omega \longrightarrow U \subset \mathbb{R}^n$ be an orientation preserving diffeomorphism onto a bounded subset U of $\mathbb{R}^n$. Suppose that $\mathcal{S} \circ \psi^{-1}$ extends via continuity to ∂U and denote by $\partial\mathcal{S}$ this continuous extension when restricted to ∂U. Then $\partial\mathcal{S}$ partitions Y into open connected components $V_1, \dots, V_k$ such that $\bigcup_{i=1}^k V_i = Y - \partial\mathcal{S}(\partial U)$.

For each $i \in \{1, \dots, k\}$, we may write $\mathcal{S}^{-1}(V_i) = \bigcup_{j=1}^{l_i} U_{ij}$ where $U_{i1}, \dots U_{il_i}$ are connected components of $\mathcal{S}^{-1}(V_i)$. Clearly, $\mathcal{S}|_{U_{ij}}$ is proper. We can therefore define the ij^{th} local skyrmion number of $\mathcal{S}$ by

$$\mathrm{SkyN}_{ij}(\mathcal{S}) \coloneqq \deg \mathcal{S}|_{U_{ij}},$$

where we understand $\deg \mathcal{S}|_{U_{ij}}$ as arising from the map $\mathcal{S}|_{U_{ij}}^{\#}\colon H_c^n(V_i) \longrightarrow H_c^n(U_{ij})$. We further define the ith skyrmion number of $\mathcal{S}$ by

$$\mathrm{SkyN}_i(\mathcal{S}) \coloneqq \begin{cases} 0, & \text{if } \mathcal{S}^{-1}(V_i) = \varnothing, \\ \sum_{j=1}^{l_i} \mathrm{SkyN}_{ij}(\mathcal{S}), & \text{otherwise.} \end{cases}$$

Lastly, we define the generalized skyrmion number of $\mathcal{S}$ by $\mathrm{SkyN}(\mathcal{S}) \in \coprod_{i=1}^{\infty} \mathbb{Z}^i$,

$$\mathrm{SkyN}(\mathcal{S}) \coloneqq (\mathrm{SkyN}_1(\mathcal{S}), \dots, \mathrm{SkyN}_k(\mathcal{S})),$$

and call $\mathcal{S}$ a Y-valued generalized skyrmion of class k.

We now state the primary topological robustness result for the generalized skyrmion number.

Theorem 3. Let Ω_z be an open subset of $\mathbb{R}^n$ for each $z \in [z_0, z_f]$ and $\mathcal{S}^z\colon \Omega_z \longrightarrow Y$ a smooth map. Let V_i^z correspond to the connected component of Y that defines the ith generalized skyrmion number of $\mathcal{S}^z$. Suppose there exists an open set $W \subset \bigcap_{z\in[z_0,z_f]} V_i^z$ and (ψ_z, F_z)-extensions of $\mathcal{S}^z$ so that

1. $F_z(X - \psi_z(\Omega_z)) \cap W = \varnothing$ for all $z \in [z_0, z_f]$ and
2. $H\colon X \times [z_0, z_f] \longrightarrow Y$, $H(x, z) = \tilde{\mathcal{S}}^z_{F_z}(x)$ is smooth,

then $\mathrm{SkyN}_i(\mathcal{S}^{z_1}) = \mathrm{SkyN}_i(\mathcal{S}^{z_2})$ for all $z_1, z_2 \in [z_0, z_f]$.

Proof: Let $\omega \in \Lambda^n(Y)$ be normalized and compactly supported in W. Then we have the following chain of equalities

$$\mathrm{SkyN}_i(\mathcal{S}^{z_1}) = \mathrm{SkyN}_\omega(\mathcal{S}^{z_1}) = \deg \tilde{\mathcal{S}}^{z_1}_{F_{z_1}} = \deg \tilde{\mathcal{S}}^{z_2}_{F_{z_2}} = \mathrm{SkyN}_\omega(\mathcal{S}^{z_2}) = \mathrm{SkyN}_i(\mathcal{S}^{z_2}),$$

where $\deg \tilde{\mathcal{S}}^{z_1}_{F_{z_1}} = \deg \tilde{\mathcal{S}}^{z_2}_{F_{z_2}}$ comes from the homotopy invariance of the degree. ■

As a powerful corollary to this, when $Y = S^2$, we may use generalizations of the (ψ, F)-extension defined by equation (3) to prove the following.

Corollary 4. Let Ω be a connected open subset of $\mathbb{R}^2$ and $\mathcal{S}\colon \Omega \times [z_0, z_f] \longrightarrow S^2$ a smooth map. Suppose $\psi\colon \Omega \longrightarrow U$ is an orientation preserving diffeomorphism onto a bounded subset U of $\mathbb{R}^2$ so that $\mathcal{S}|_{\Omega\times\{z\}}\circ\psi^{-1}$ extends via continuity to ∂U for all $z \in [z_0, z_f]$. Let V_i^z correspond to the connected component of Y that defines the ith generalized skyrmion number of $\mathcal{S}^z \coloneqq \mathcal{S}|_{\Omega\times\{z\}}$. Suppose there exists an open set $W \subset \bigcap_{z\in[z_0,z_f]} V_i^z$, then $\mathrm{SkyN}_i(\mathcal{S}^{z_1}) = \mathrm{SkyN}_i(\mathcal{S}^{z_2})$ for all $z_1, z_2 \in [z_0, z_f]$.

Proof: Here, we give a somewhat qualitative proof. Notice first that ∂U is homeomorphic to a disjoint union of circles $\partial U \cong \coprod_{j=1}^m S^1$. Let φ be any inverse stereographic projection map. We construct a

$(\varphi \circ \psi, F)$-extension in the following way. For each $j = 1, \ldots, m$, pick a homeomorphism $\mathcal{C}_j \colon S^1 \longrightarrow \mathbb{R}^2$ that parameterizes the corresponding circle of ∂U. Then $\varphi \circ \mathcal{C}_j$ is a circle in S^2 which partitions S^2 into two components. By the connectedness of Ω, $\varphi(\psi(\Omega))$ is contained in exactly one of these components. Now, construct a continuous homotopy $H_j \colon S^1 \times [0,1] \longrightarrow S^2$ so that $H_j(s,0) = \varphi(\mathcal{C}_j(s))$, $H_j(s,1)$ is some constant c_j, $H_j|_{S^1 \times [0,1)}$ is injective, and that the image of H_j is the union of the image of $\varphi \circ \mathcal{C}_j$ and the connected component of S^2 carved out by $\varphi \circ \mathcal{C}_j$ not containing $\varphi(\psi(\Omega))$.

Now let $p \in B_r(0) \cap S^2 = W' \subset W$. Note, by definition, the curve $\partial\mathcal{S}^z \circ \mathcal{C}_j$, where as usual $\partial\mathcal{S}^z$ denotes the continuous extension of $\mathcal{S}^z \circ \psi^{-1}$ restricted to ∂U, does not intersect W and hence W'. Therefore, we may, for each j, define $F_j^z \colon H_j(S^1 \times [0,1]) \longrightarrow S^2$

$$F_j^z(H_j(s,t)) = \frac{(1-t)(\partial\mathcal{S}^z \circ \mathcal{C}_j)(s) - tp}{\|(1-t)(\partial\mathcal{S}^z \circ \mathcal{C}_j)(s) - tp\|}$$

so that $F_j^z(H_j(S^1 \times [0,1])) \cap W' = \varnothing$. By defining F^z by $F^z|_{H_j(S^1 \times [0,1])} = F_j^z$, we have constructed $(\varphi \circ \psi, F^z)$-extensions of $\mathcal{S}^z$ which satisfy the assumptions of theorem 3. ∎

Corollary 4 ties our abstract mathematical results to the qualitative discussions presented in the main text, namely that as long as there exists some overlap of a given connected component as it transforms, then topological information can be relayed from one state to another.

2 Supplementary Note

2.1 The Algebraic Perspective

In this section, we provide an alternative interpretation of the generalized skyrmion number from an algebraic perspective. Before turning to the technical details, we note several key qualitative differences between the differential-geometric and algebraic perspectives. The former is computationally more convenient and therefore better suited to engineering applications, where computing the generalized skyrmion number from experimental data is of central importance. The latter, by contrast, is conceptually cleaner and thus more naturally suited to theoretical development. Given the strong engineering focus of this work, we develop the former in greater detail and only briefly discuss the latter.

The algebraic analogue of de Rham cohomology with compact support is relative cohomology. Thus, if $\Omega \subset \mathbb{R}^n$ is a closed set and $f \colon \Omega \to Y$ is continuous, then f induces a map

$$f^* \colon H^\bullet\big(Y, f(\partial\Omega)\big) \longrightarrow H^\bullet(\Omega, \partial\Omega)$$

in relative cohomology. The generalized skyrmion number is then given by the induced map in degree n, namely

$$f^* \colon H^n\big(Y, f(\partial\Omega)\big) \longrightarrow H^n(\Omega, \partial\Omega).$$

More concretely, in the case $\Omega = \overline{B_1(0)}$ and $Y = S^2$, if the pair $(Y, f(\partial\Omega))$ is a good pair [4], as is the case in all examples considered in this work, then

$$H^2(S^2, f(\partial\Omega)) \cong H^2(S^2/f(\partial\Omega)).$$

Moreover, $S^2/f(\partial\Omega)$ is just a wedge of spheres, one for each connected component of $S^2 - f(\partial\Omega)$. As such

$$H^2(S^2/f(\partial\Omega)) \cong H^2(\vee^k S^2) \cong \mathbb{Z}^k,$$

where $k = |\pi_0(S^2 - f(\partial\Omega))|$, so that

$$f^* \colon H^2(S^2/f(\partial\Omega)) \cong \mathbb{Z}^k \longrightarrow H^2(\Omega, \partial\Omega) \cong \mathbb{Z}$$

is described by k integers once the isomorphisms are fixed. Taking the standard basis on both sides gives the algebraic definition of the generalized skyrmion numbers.

One key advantage of adopting the algebraic approach is that other topological invariants of f, defined through induced maps in different degrees, are easily identified. More generally, any pair of maps $f, g\colon (\Omega, \partial\Omega) \longrightarrow (Y, V)$ which are homotopic through maps of pairs induce identical maps on the associated long exact sequence in cohomology,

$$\begin{array}{ccccccccccc}
\cdots & \longrightarrow & H^n(V) & \longrightarrow & H^{n+1}(Y,V) & \longrightarrow & H^{n+1}(Y) & \longrightarrow & H^{n+1}(V) & \longrightarrow & \cdots \\
 & & \downarrow{\scriptstyle f^*=g^*} & & \downarrow{\scriptstyle f^*=g^*} & & \downarrow{\scriptstyle f^*=g^*} & & \downarrow{\scriptstyle f^*=g^*} & & \\
\cdots & \longrightarrow & H^n(\partial\Omega) & \longrightarrow & H^{n+1}(\Omega,\partial\Omega) & \longrightarrow & H^{n+1}(\Omega) & \longrightarrow & H^{n+1}(\partial\Omega) & \longrightarrow & \cdots .
\end{array}$$

Thus, this collection of maps f^* can be regarded as a "topological signature" of the map f, defining various topological invariants that capture phenomena of different dimensions.

For example, in optical polarization fields, the generalized skyrmion number is sensitive only to two-dimensional information. As such, if you consider the purely radial polarization field $\mathcal{S}_n\colon \Omega \longrightarrow S^2$ defined on the closed annulus $\Omega = B_R(0) - B_\varepsilon(0)$ given by

$$\mathcal{S}_n(r,\theta) = (\cos n\theta, \sin n\theta, 0),$$

such a field defines a generalized skyrmion number of $(0,0)$ despite being topologically distinct. On the other hand, when considering the long exact sequence in cohomology above, the situation is quite different. Let S_{eq} denote the equator of S^2. We then have

$$\begin{array}{ccccccccc}
0 & \longrightarrow & H^1(S_{\text{eq}}) \cong \mathbb{Z} & \longrightarrow & H^2(S^2, S_{\text{eq}}) \cong \mathbb{Z}^2 & \longrightarrow & H^2(S^2) \cong \mathbb{Z} & \longrightarrow & 0 \\
 & & \downarrow{\scriptstyle \mathcal{S}_n^*} & & \downarrow{\scriptstyle \mathcal{S}_n^*} & & \downarrow{\scriptstyle \mathcal{S}_n^*} & & \\
H^1(\Omega) \cong \mathbb{Z} & \longrightarrow & H^1(\partial\Omega) \cong \mathbb{Z}^2 & \longrightarrow & H^2(\Omega,\partial\Omega) \cong \mathbb{Z} & \longrightarrow & 0 & \longrightarrow & 0.
\end{array}$$

Here, the maps $\mathcal{S}_n^*\colon H^1(S_{\text{eq}}) \to H^1(\partial\Omega)$ capture the lower-dimensional topological differences between the fields $\mathcal{S}_n$.

3 Supplementary Figures

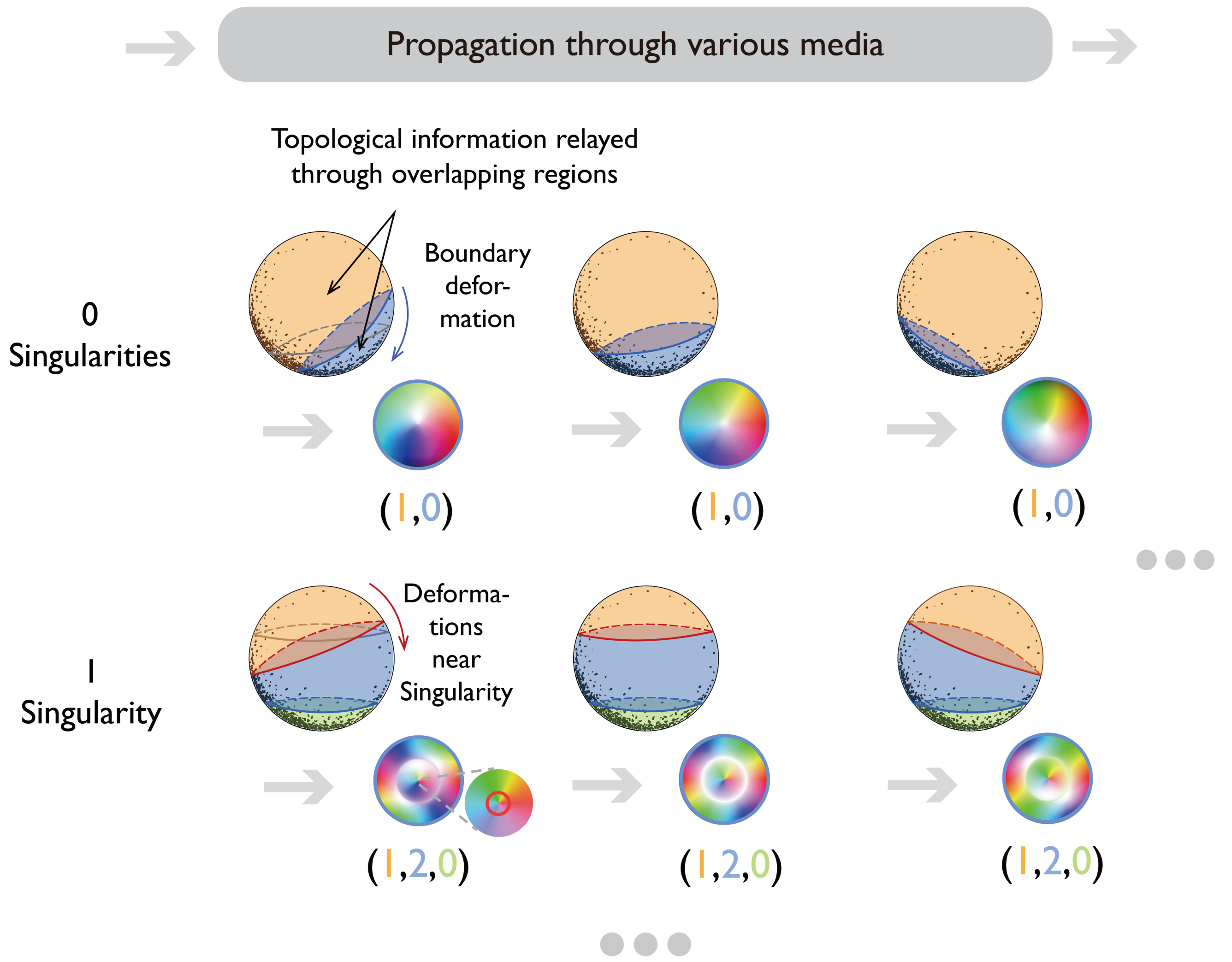

Supplementary Fig. 1: **Topological robustness of the generalized skyrmion number.** Various fields in propagation and the corresponding components carved out by their boundaries. In each case, the smoothness of propagation gives rise to overlaps persistent throughout, which allows topological information to be relayed from the initial field to the final field. This then guarantees the preservation of the generalized skyrmion number. The top row shows a scenario without singularities, and the bottom row a scenario with a single singularity.

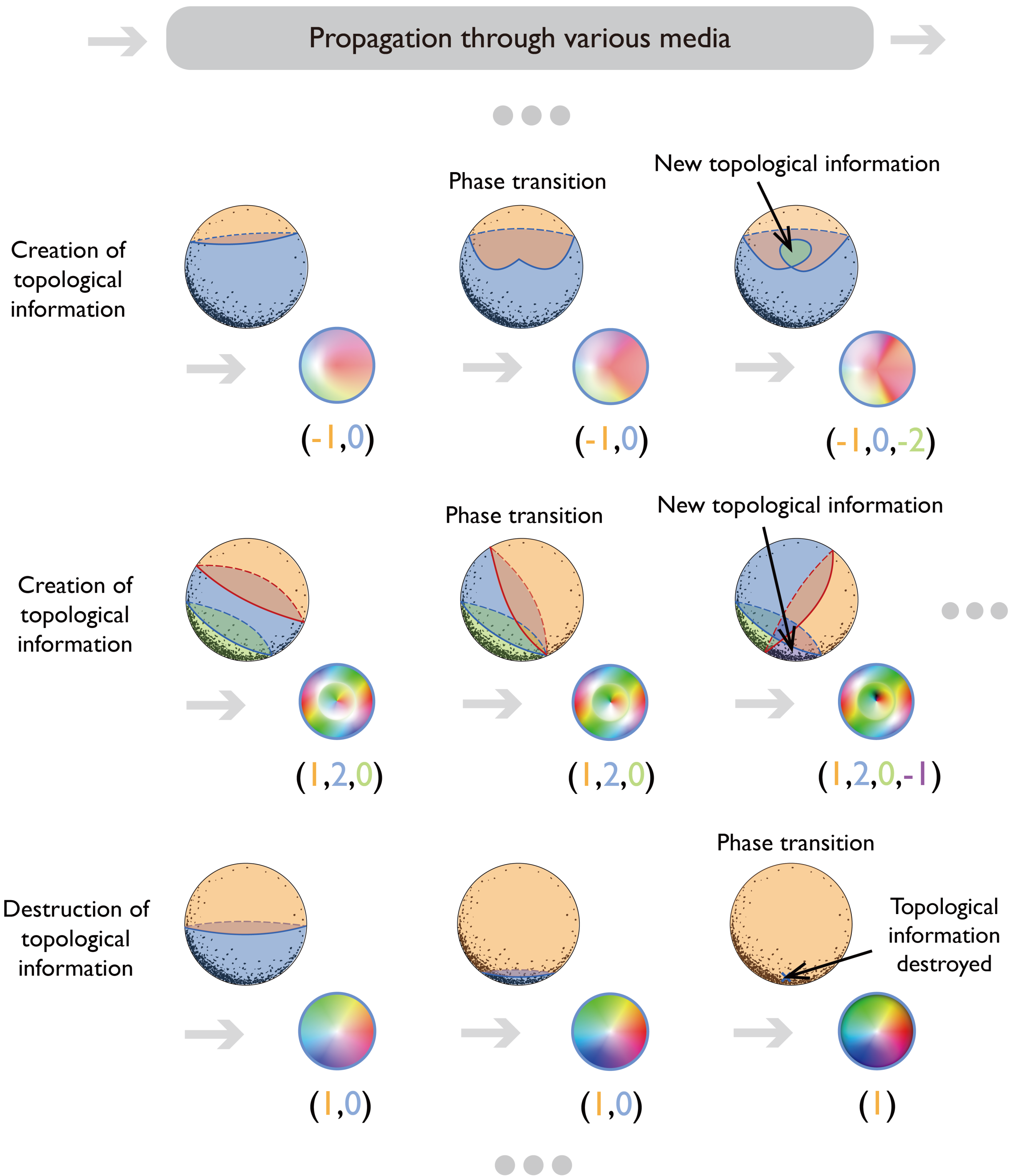

Supplementary Fig. 2: **Creation and destruction of topological information through phase transitions.** Various topological phase transitions that can occur which lead to the creation and destruction of topological information. (Top) Topological information is created through self-intersection of the outer boundary. (Middle) Topological information is created through the intersection of the outer boundary and the "boundary" of the singularity. (Bottom) Topological information is destroyed as the outer boundary collapses into a single point.

References

[1] Wang, A. A. *et al.* Topological protection of optical skyrmions through complex media. *Light: Science & Applications* **13**, 314 (2024).

[2] Weintraub, S. H. *Differential Forms: Theory and Practice* (Academic Press, 2014), 2nd edn.

[3] Naber, G. L. *Topology, Geometry and Gauge fields Interactions.* Applied Mathematical Sciences (Springer, 2011), 2nd edn.

[4] Hatcher, A. *Algebraic Topology* (Cambridge University Press, 2001).
