# Supplementary material for: Redefining topological robustness in optical polarization fields through a generalized skyrmion number
Source: Nat Commun. 2026 Jul 10;17:8539. doi: 10.1038/s41467-026-75120-7 (PMC13483919; doi:10.1038/s41467-026-75120-7)
Supplement: Supplementary file 2 [file 41467_2026_75120_MOESM2_ESM.pdf]

nature portfolio

Peer Review File

Redefining topological robustness in optical polarization fields through a generalized skyrmion number

Corresponding Author: Professor Chao He

This manuscript has been previously reviewed at another journal. This document only contains information relating to versions considered at Nature Communications.

This file contains all reviewer reports in order by version, followed by all author rebuttals in order by version.

Version 0:

Reviewer comments:

Reviewer #3

(Remarks to the Author)
Dear editor,
Dear authors

I had the pleasure of reviewing your article: Redefining topological robustness in optical polarization fields through a generalized skyrmion number. In general I do find the approach robust and the results shown therein scientifically sound. Introducing a new formalism and extending it to optical polarization is innovative and, in my opinion it will provide a contribution for future works on this field. Considering the relevance that topological protection is offering towards quantum communications and more I do find the works of this paper relevant for a broader community, such as the one Nature Communications represents. Therefore I do recommend publication of the article.

As a comment, I do find the paper somehow difficult to navigate through if you are not a specialist. I would recommend a figure that catches the principle and that is self contained (even a non-specialist should grasp the concept).

In conclusion I recommend publication of this work and I congratulate you on your results.
Regards

Reviewer #4

(Remarks to the Author)
This manuscript presents a theoretical generalization of the Skyrmion number for fields, particularly optical polarization fields, that do not satisfy the strict boundary conditions, i.e., constant along the circle at infinity, required for traditional compactification. By leveraging the de Rham cohomology of compactly supported forms, the authors define a new topological invariant valued in {Zi}. This generalized Skyrmion number assigns a tuple of integers to a field based on how its boundary curves partitions the target Poincaré sphere. The theoretical framework is developed rigorously in the Supplementary Information, and experimental results on propagating beams demonstrate the robustness of this new invariant in scenarios where the traditional Skyrmion number fails. I recommend acceptance after the following issues have been addressed.
Major comments and questions
1. The definition of the generalized Skyrmion number relies on the boundary curve ∂S(∂Ω) partitioning the target space Y (e.g., the Poincaré sphere S^2) into connected components Vi. The topological number for a component Vi is non-zero only if its preimage S^{−1}(Vi) is non-empty. Consider two fundamentally different fields: a purely radial polarization field where the polarization direction winds once around a circle, and a field where it winds twice around a circle. Both fields map

entirely to the equator of the Poincaré sphere. Consequently, the northern and southern hemispheres (V1, V2) are not covered, yielding a generalized Skyrmion number of (0, 0) for both fields. Intuitively, these two fields are topologically distinct (they have winding numbers of 1 and 2, respectively). Could the authors please clarify how the generalized Skyrmion number, as defined, captures this type of topological information that resides entirely on the cut itself (the equator) rather than in the regions it defines? Does this represent a fundamental limitation of the invariant, or is there a way to recover this information within the proposed framework?
2. The experimental verification and primary examples (e.g., the waveguide mode)
focus on fields with a minimal number of singularities, resulting in generalized Skyrmion numbers that are relatively short tuples. For more complex, e.g., superpositions of many higher-order Hermite-Gaussian or Laguerre-Gaussian modes, the number of phase and polarization singularities can be very large. This would lead to a highly complicated boundary curve on the Poincaré sphere and partitioning it into numerous connected components would produce a very long integer tuple. Does the proposed method remain computationally feasible and robust for such complex fields? How does the condition for topological protection scale with the number of components? As the number of connected components becomes large, is it not increasingly likely that small components will be ephemeral or that the required common overlap will vanish under small perturbations, thereby limiting the practical stability of very long tuples?
3. The manuscript successfully generalizes the global Skyrmion number to a tuple of
integers derived from the field's behavior in different target-space regions (partition of the Poincare shpher). However, a simple field like a uniformly linearly polarized vortex beam possesses two distinct topological features, i.e., a trivial Skyrmion number of 0 (as its polarization is constant) and a non-trivial orbital angular momentum characterized by a vortex winding number. The proposed generalized Skyrmion number based on polarization (Stokes field) would only capture the first feature. Is there a vision for extending this cohomological framework to create a more unified topological indicator that could simultaneously encode information from different physical quantities (e.g., both polarization and phase) within a single tuple or other algebraic structure? This would be a powerful step toward fully characterizing the topological complexity of structured light fields.
4. The paper tackles the challenge of defining topological numbers for fields that are
not compactifiable in real space. A complementary approach, particularly relevant for propagating fields, would be to work in momentum space. The angular spectrum of a propagating monochromatic field is naturally confined to a sphere of with radius equal to the wavenumber k (the k-sphere), which is a compact manifold. How does the generalized Skyrmion number defined in real space compare to a topological invariant (e.g., a Chern number) that could be defined directly on the k-sphere from the angular spectrum? Would an angular spectrum approach potentially simplify the mathematics by circumventing the need for compactification and extensions, as the domain is already compact?

Version 1:

Reviewer comments:

Reviewer #4

(Remarks to the Author)
The authors' response is satisfactory. In particular, I find the introduction of the long exact sequence in cohomology to be a valuable perspective, as it clarifies how topological information that is missing from the original generalized Skyrmion number framework,e.g., the winding number of a radially-polarzied field confined to the equator, can be systematically recovered.

Open Access This Peer Review File is licensed under a Creative Commons Attribution 4.0 International License, which permits use, sharing, adaptation, distribution and reproduction in any medium or format, as long as you give appropriate credit to the original author(s) and the source, provide a link to the Creative Commons license, and indicate if changes were made.
In cases where reviewers are anonymous, credit should be given to 'Anonymous Referee' and the source.
The images or other third party material in this Peer Review File are included in the article's Creative Commons license, unless indicated otherwise in a credit line to the material. If material is not included in the article's Creative Commons license and your intended use is not permitted by statutory regulation or exceeds the permitted use, you will need to obtain permission directly from the copyright holder.
To view a copy of this license, visit https://creativecommons.org/licenses/by/4.0/

Responses to Referee's comments (Referee #3)

I had the pleasure of reviewing your article: Redefining topological robustness in optical polarization fields through a generalized skyrmion number. In general I do find the approach robust and the results shown therein scientifically sound. Introducing a new formalism and extending it to optical polarization is innovative and, in my opinion it will provide a contribution for future works on this field. Considering the relevance that topological protection is offering towards quantum communications and more I do find the works of this paper relevant for a broader community, such as the one Nature Communications represents. Therefore I do recommend publication of the article.

We sincerely thank the reviewer for their positive assessment of our work and for their thoughtful and encouraging comments. We are pleased that the reviewer finds the proposed formalism both robust and scientifically sound, and that its extension to optical polarization fields is viewed as innovative and of potential value to future research in the area.

We also appreciate the insightful remark regarding the relevance of topological protection to quantum communications and related fields. This is an important perspective, and in response we have incorporated additional references (see below) in the manuscript to better highlight these broader connections and potential applications.

"It is not surprising, then, that the discovery of a new topological number is often the central catalyst for advancements across multiple scientific disciplines, as evidenced by the appearance of skyrmions in diverse fields ranging from the study of Bose-Einstein condensates [3] and magnetic spin textures [4,5] in condensed matter physics to optics and photonics [6-26], quantum technologies [Nature Photonics 18, 258-266 (2024), Nature Communications 16, 2934 (2025), Nature Physics 21, 1462-1468 (2025)], cosmology [30], fluid dynamics [31], soft matter physics [32], acoustics [33,34], and more [35,36]."

Thank you again for your careful reading and support of our work.

As a comment, I do find the paper somehow difficult to navigate through if you are not a specialist. I would recommend a figure that catches the principle and that is self contained (even a non-specialist should grasp the concept).

In conclusion I recommend publication of this work and I congratulate you on your results.

Department of Engineering Science
Parks Road, Oxford OX1 3PJ
Email: aloysius.wang@gmail.com; chao.he@eng.ox.ac.uk

We thank the reviewer for this helpful suggestion and for their positive overall assessment of our work. We agree that improving accessibility for non-specialist readers is important, particularly given the broader relevance of the topic.

In response, we have updated our figures to include a more self-contained explanation of the generalized skyrmion number, as shown below. We hope this addresses your concerns and clarifies the definition of the generalized skyrmion number for non-specialist readers.

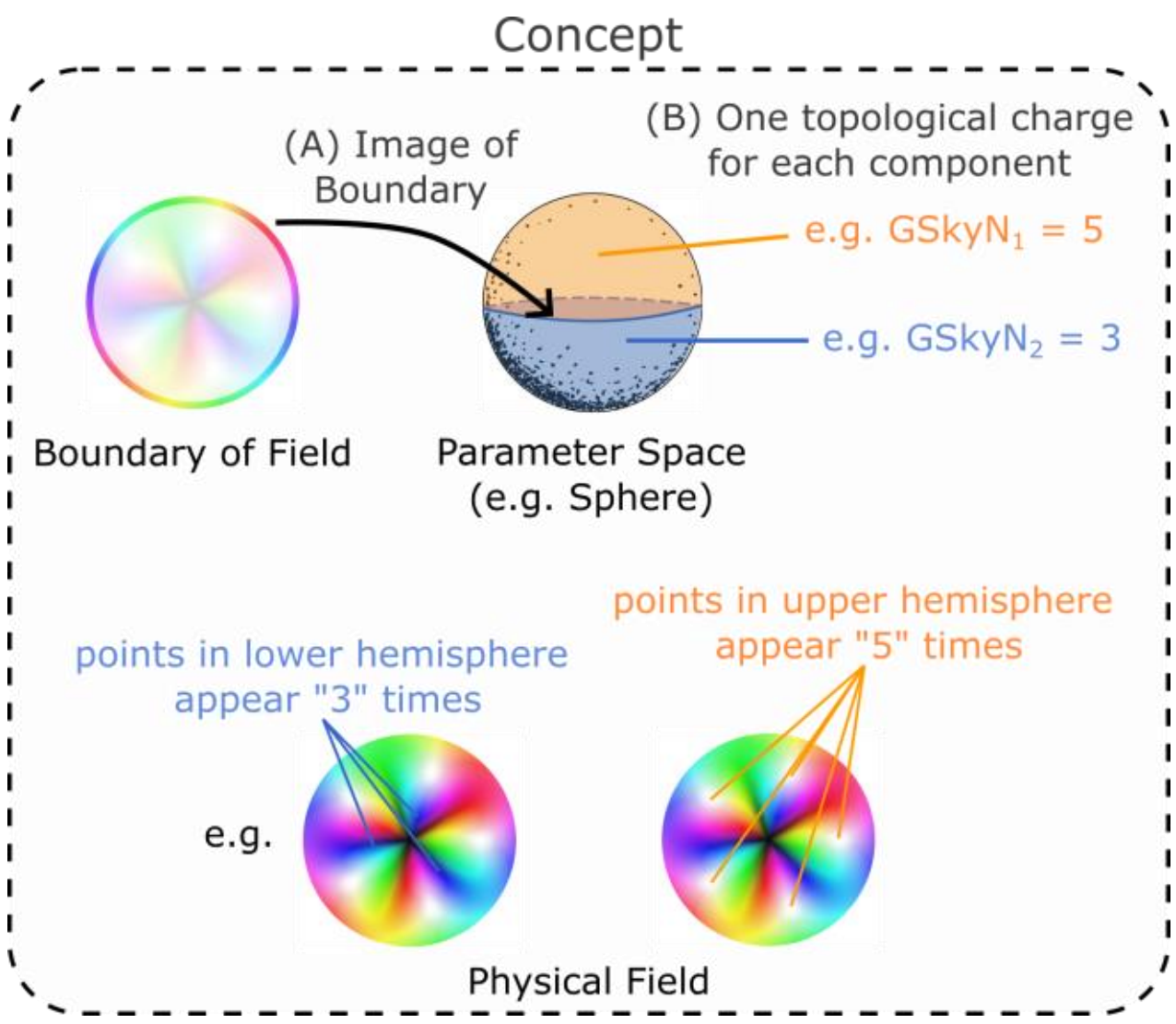

Fig. 2: Concept of the generalized skyrmion number. The image of the boundary of the S^2-valued field defines a curve in parameter space. A single topological number can then be assigned to each component carved out by this boundary curve, representing the signed number of times the field "wraps" around that component, with orientation-preserving and orientation-reversing coverings contributing positively and negatively, respectively.

We thank the reviewer again for the valuable feedback and for their recommendation of our work.

Department of Engineering Science
Parks Road, Oxford OX1 3PJ
Email*:* aloysius.wang@gmail.com*;* chao.he@eng.ox.ac.uk

Responses to Referee's comments (Referee #4)

This manuscript presents a theoretical generalization of the Skyrmion number for fields, particularly optical polarization fields, that do not satisfy the strict boundary conditions, i.e., constant along the circle at infinity, required for traditional compactification. By leveraging the de Rham cohomology of compactly supported forms, the authors define a new topological invariant valued in {Zi}. This generalized Skyrmion number assigns a tuple of integers to a field based on how its boundary curves partitions the target Poincaré sphere. The theoretical framework is developed rigorously in the Supplementary Information, and experimental results on propagating beams demonstrate the robustness of this new invariant in scenarios where the traditional Skyrmion number fails. I recommend acceptance after the following issues have been addressed.

We thank the reviewer for their careful reading of our manuscript and for the clear and thoughtful summary of our work. We appreciate their recognition of both the theoretical development and the experimental validation presented, as well as their recommendation for publication.

We are grateful for the reviewer's feedback and have addressed the points raised below.

Major comments and questions

1. The definition of the generalized Skyrmion number relies on the boundary curve ∂S(∂Ω) partitioning the target space Y (e.g., the Poincaré sphere S^2) into connected components Vi. The topological number for a component Vi is non-zero only if its preimage S^{−1}(Vi) is non-empty. Consider two fundamentally different fields: a purely radial polarization field where the polarization direction winds once around a circle, and a field where it winds twice around a circle. Both fields map entirely to the equator of the Poincaré sphere. Consequently, the northern and southern hemispheres (V1, V2) are not covered, yielding a generalized Skyrmion number of (0, 0) for both fields. Intuitively, these two fields are topologically distinct (they have winding numbers of 1 and 2, respectively). Could the authors please clarify how the generalized Skyrmion number, as defined, captures this type of topological information that resides entirely on the cut itself (the equator) rather than in the regions it defines? Does this represent a fundamental limitation of the invariant, or is there a way to recover this information within the proposed framework?

Thank you for your insightful comment and example. You are correct that the generalized skyrmion number is unable to detect such topological differences, as it arises as an induced

Department of Engineering Science
Parks Road, Oxford OX1 3PJ
Email: aloysius.wang@gmail.com; chao.he@eng.ox.ac.uk

map in second cohomology and is therefore sensitive only to two-dimensional information. However, the topological distinctions between the radial polarization fields you describe can still be detected within a broader algebraic framework, which we have avoided introducing in the main text due to its technical nature. We have now added an additional section to the Supplementary Information addressing this point, which we have included below as a screenshot for your convenience.

2 The Algebraic Perspective

In this section, we provide an alternative interpretation of the generalized skyrmion number from an algebraic perspective. Before turning to the technical details, we note several key qualitative differences between the differential-geometric and algebraic perspectives. The former is computationally more convenient and therefore better suited to engineering applications, where computing the generalized skyrmion number from experimental data is of central importance. The latter, by contrast, is conceptually cleaner and thus more naturally suited to theoretical development. Given the strong engineering focus of this work, we develop the former in greater detail and only briefly discuss the latter.

The algebraic analogue of de Rham cohomology with compact support is relative cohomology. Thus, if $\Omega \subset \mathbb{R}^n$ is a closed set and $f\colon \Omega \to Y$ is continuous, then f induces a map

$$f^*\colon H^\bullet\big(Y, f(\partial\Omega)\big) \longrightarrow H^\bullet(\Omega, \partial\Omega)$$

in relative cohomology. The generalized skyrmion number is then given by the induced map in degree n, namely

$$f^*\colon H^n\big(Y, f(\partial\Omega)\big) \longrightarrow H^n(\Omega, \partial\Omega).$$

More concretely, in the case $\Omega = \overline{B_1(0)}$ and $Y = S^2$, if the pair $(Y, f(\partial\Omega))$ is a good pair [4], as is the case in all examples considered in this work, then

$$H^2(S^2, f(\partial\Omega)) \cong H^2(S^2/f(\partial\Omega)).$$

Moreover, $S^2/f(\partial\Omega)$ is just a wedge of spheres, one for each connected component of $S^2 - f(\partial\Omega)$. As such

$$H^2(S^2/f(\partial\Omega)) \cong H^2(\vee^k S^2) \cong \mathbb{Z}^k,$$

where $k = |\pi_0(S^2 - f(\partial\Omega))|$, so that

$$f^*\colon H^2(S^2/f(\partial\Omega)) \cong \mathbb{Z}^k \longrightarrow H^2(\Omega, \partial\Omega) \cong \mathbb{Z}$$

is described by k integers once the isomorphisms are fixed. Taking the standard basis on both sides gives the algebraic definition of the generalized skyrmion numbers.

6

Department of Engineering Science
Parks Road, Oxford OX1 3PJ
Email: aloysius.wang@gmail.com; chao.he@eng.ox.ac.uk

One key advantage of adopting the algebraic approach is that other topological invariants of f, defined through induced maps in different degrees, are easily identified. More generally, any pair of maps $f, g\colon (\Omega, \partial\Omega) \longrightarrow (Y, V)$ which are homotopic through maps of pairs induce identical maps on the associated long exact sequence in cohomology,

$$\begin{array}{ccccccccc} \cdots \longrightarrow & H^n(V) & \longrightarrow & H^{n+1}(Y,V) & \longrightarrow & H^{n+1}(Y) & \longrightarrow & H^{n+1}(V) & \longrightarrow \cdots \\ & \downarrow{\scriptstyle f^*=g^*} & & \downarrow{\scriptstyle f^*=g^*} & & \downarrow{\scriptstyle f^*=g^*} & & \downarrow{\scriptstyle f^*=g^*} & \\ \cdots \longrightarrow & H^n(\partial\Omega) & \longrightarrow & H^{n+1}(\Omega,\partial\Omega) & \longrightarrow & H^{n+1}(\Omega) & \longrightarrow & H^{n+1}(\partial\Omega) & \longrightarrow \cdots . \end{array}$$

Thus, this collection of maps f^* can be regarded as a "topological signature" of the map f, defining various topological invariants that capture phenomena of different dimensions.

For example, in optical polarization fields, the generalized skyrmion number is sensitive only to two-dimensional information. As such, if you consider the purely radial polarization field $\mathcal{S}_n\colon \Omega \longrightarrow S^2$ defined on the closed annulus $\Omega = B_R(0) - B_\varepsilon(0)$ given by

$$\mathcal{S}_n(r,\theta) = (\cos n\theta, \sin n\theta, 0),$$

such a field defines a generalized skyrmion number of $(0,0)$ despite being topologically distinct. On the other hand, when considering the long exact sequence in cohomology above, the situation is quite different. Let S_{eq} denote the equator of S^2. We then have

$$\begin{array}{ccccccccc} 0 & \longrightarrow & H^1(S_{\text{eq}}) \cong \mathbb{Z} & \longrightarrow & H^2(S^2, S_{\text{eq}}) \cong \mathbb{Z}^2 & \longrightarrow & H^2(S^2) \cong \mathbb{Z} & \longrightarrow & 0 \\ & & \downarrow{\scriptstyle \mathcal{S}_n^*} & & \downarrow{\scriptstyle \mathcal{S}_n^*} & & \downarrow{\scriptstyle \mathcal{S}_n^*} & & \\ H^1(\Omega) \cong \mathbb{Z} & \longrightarrow & H^1(\partial\Omega) \cong \mathbb{Z}^2 & \longrightarrow & H^2(\Omega, \partial\Omega) \cong \mathbb{Z} & \longrightarrow & 0 & \longrightarrow & 0. \end{array}$$

Here, the maps $\mathcal{S}_n^*\colon H^1(S_{\text{eq}}) \to H^1(\partial\Omega)$ capture the lower-dimensional topological differences between the fields $\mathcal{S}_n$.

From the above, it may be seen that while the generalized skyrmion number itself cannot detect such topological information, the invariant $f^*\colon H^1\big(f(\partial\Omega)\big) \longrightarrow H^1(\partial\Omega)$ can. We note three important additional caveats: (1) The field you have defined possesses a singularity at $r = 0$. Fields without such a singularity, but with the same radial boundary condition at infinity, must necessarily extend into either the upper or lower hemisphere in order to close the boundary loop. As such, they define generalized skyrmions of degree $(n, 0)$ or $(0, -n)$. In this case, the winding number at infinity is directly reflected in the generalized skyrmion number. Thus, the fact that the generalized skyrmion number cannot distinguish between such fields is due to the singularity at $r = 0$.

(2) However, homotopies within this generalized skyrmion framework can distinguish between such radial fields. Indeed, any homotopy from, say, the field of order 2 to that of order 1 must either collapse the boundary curve, leading to a reduction in the number of generalized skyrmion components, or destroy the symmetry of the boundary curve, resulting in an increase in the number of such components.

(3) The field you have given can actually be assigned a third topological invariant as its image lies entirely in S_{eq}. Thus, it defines a non-trivial map $f^*\colon H^1\big(S_{\text{eq}}\big) \longrightarrow H^1(\Omega)$.

Department of Engineering Science
Parks Road, Oxford OX1 3PJ
Email: aloysius.wang@gmail.com; chao.he@eng.ox.ac.uk

However, as the property of lying in S_{eq} is not stable under perturbations, this invariant will be less stable than the invariant $f^*: H^1(f(\partial\Omega)) \longrightarrow H^1(\partial\Omega)$ defined above.

2. The experimental verification and primary examples (e.g., the waveguide mode) focus on fields with a minimal number of singularities, resulting in generalized Skyrmion numbers that are relatively short tuples. For more complex, e.g., superpositions of many higher-order Hermite-Gaussian or Laguerre-Gaussian modes, the number of phase and polarization singularities can be very large. This would lead to a highly complicated boundary curve on the Poincaré sphere and partitioning it into numerous connected components would produce a very long integer tuple. Does the proposed method remain computationally feasible and robust for such complex fields? How does the condition for topological protection scale with the number of components? As the number of connected components becomes large, is it not increasingly likely that small components will be ephemeral or that the required common overlap will vanish under small perturbations, thereby limiting the practical stability of very long tuples?

Thank you for your insightful comments regarding the practical stability of the generalized skyrmion number. You are correct to note that a field with many singularities will be assigned a large tuple of integers, reflecting the complexity and intersections of the associated boundary curves. This is, in fact, one of the key advantages of the generalized skyrmion number as a means of increasing information density.

From a computational perspective, since each generalized skyrmion number is obtained via an integral, and assuming the relevant kernels are known a priori, the time required to compute the full generalized skyrmion number is $O(kn^2)$ where k is the number of generalized skyrmion numbers and n is the linear dimension of the data. This is no different from computing the corresponding information for k conventional skyrmions of linear dimension n. If, on the other hand, the kernels are not known a priori, determining them requires analyzing the intersections of the boundary curves, which scales as p^2n^2, where p is the number of singularities. However, if an engineering lookup table is employed, this cost can be reduced to $O(n)$ so that the dominant cost remains the integration. As such, depending on the available a priori information and the engineering methods employed, the computational cost of analyzing complex fields can be significantly reduced, making such approaches feasible for real-world applications.

The topological protection of each component is likely to scale with its area, therefore, one expects it to scale inversely with the number of components. Nonetheless, one expects that, at scales relevant to nanophotonics, the generalized skyrmion number is a

Department of Engineering Science
Parks Road, Oxford OX1 3PJ
Email: aloysius.wang@gmail.com; chao.he@eng.ox.ac.uk

fairly stable quantity, whereas in the far field not all generalized skyrmion numbers are preserved. Note also that with recent work encoding skyrmionic structures into the optical anisotropy of retarder fields, this framework of generalized skyrmions can be used as a strategy for very-high-density information storage. In this setting, the fact that many distinct singularities can interact to produce a large number of tuples, together with the intrinsic stability of matter fields (i.e., without concerns arising from propagation), suggests that alternative applications of the generalized skyrmion number hold significant potential.

3. The manuscript successfully generalizes the global Skyrmion number to a tuple of integers derived from the field's behavior in different target-space regions (partition of the Poincare shpher). However, a simple field like a uniformly linearly polarized vortex beam possesses two distinct topological features, i.e., a trivial Skyrmion number of 0 (as its polarization is constant) and a non-trivial orbital angular momentum characterized by a vortex winding number. The proposed generalized Skyrmion number based on polarization (Stokes field) would only capture the first feature. Is there a vision for extending this cohomological framework to create a more unified topological indicator that could simultaneously encode information from different physical quantities (e.g., both polarization and phase) within a single tuple or other algebraic structure? This would be a powerful step toward fully characterizing the topological complexity of structured light fields.

Thank you for your insightful suggestions. As mentioned above, we have further developed the theory of generalized skyrmions in a more algebraic direction, which accomplishes precisely what you have suggested. Additionally, we have another paper in preparation that develops a framework for topological field engineering through projections, extending the cohomological framework to treat phase and polarization simultaneously. We would be happy to provide further details if the reviewer is interested.

4. The paper tackles the challenge of defining topological numbers for fields that are not compactifiable in real space. A complementary approach, particularly relevant for propagating fields, would be to work in momentum space. The angular spectrum of a propagating monochromatic field is naturally confined to a sphere of with radius equal to the wavenumber k (the k-sphere), which is a compact manifold. How does the generalized Skyrmion number defined in real space compare to a topological invariant (e.g., a Chern number) that could be defined directly on the k-sphere from the angular spectrum? Would an angular spectrum approach potentially simplify the mathematics

Department of Engineering Science
Parks Road, Oxford OX1 3PJ
Email: aloysius.wang@gmail.com; chao.he@eng.ox.ac.uk

by circumventing the need for compactification and extensions, as the domain is already compact?

Thank you for your insightful comments. First, the main advantage of working in real space rather than k-space is twofold. The first is the ease of measurement: real-space data are directly accessible from a camera, without the need for additional components. On the other hand, accessing data in k-space (particularly when the entire sphere is considered, requiring both forward- and backward-propagating waves to be taken into account) is more challenging and thus less suitable for real-world applications. Moreover, evanescent components will quickly decay and become inaccessible after short distances. The second is the ease of generation, as controlling the coefficients over the entire sphere in k-space is a challenging task, particularly when evanescent and backward-propagating waves must also be manipulated. Nonetheless, an approach based on k-space is worth exploring as an alternative and may offer greater stability due to its inherently compact nature.

On the other hand, the Chern number arises in settings where periodicity (spatial or temporal) allows the system to be described in k-space via Bloch's theorem or its generalizations. In such cases, energy or quasi-energy bands are defined over a compact parameter space (e.g., the Brillouin zone). Here, the wavevector k should be distinguished from the angular-spectrum wavevector used in optical field decompositions; in the present context, k denotes the quasi-momentum, i.e., the Fourier parameter associated with the underlying periodicity. In this case, the topological invariant arises from the classification of principal $U(1)$-bundles over the torus, and is of a fundamentally different origin from the skyrmion number. Lastly, when spatial periodicity is present, T^2 compactifiability arises, and a skyrmion number can be defined alongside the Chern number. In this setting, it may be worthwhile to investigate similarities and differences in their stability, which we leave for future work.

We thank the reviewer again for the valuable feedback and for their recommendation of our work.

Department of Engineering Science
Parks Road, Oxford OX1 3PJ
Email: aloysius.wang@gmail.com; chao.he@eng.ox.ac.uk

Responses to Referee’s comments (Referee #4)

Reviewer #4 (Remarks to the Author):

The authors’ response is satisfactory. In particular, I find the introduction of the long exact sequence in cohomology to be a valuable perspective, as it clarifies how topological information that is missing from the original generalized Skyrmion number framework,e.g., the winding number of a radially-polarzied field confined to the equator, can be systematically recovered.

We thank the reviewer for their positive assessment of our revision. We are pleased that the introduction of the long exact sequence in cohomology is viewed as a valuable perspective. We appreciate the reviewer’s thoughtful feedback and encouragement.

Department of Engineering Science
Parks Road, Oxford OX1 3PJ
Email: aloysius.wang@gmail.com; chao.he@eng.ox.ac.uk
